# Mendelian randomization study highlights the role of hematological traits on Type-2 diabetes mellitus in African ancestry individuals

**DOI:** 10.3389/fphar.2025.1436972

**Published:** 2025-03-31

**Authors:** Chisom Soremekun, Daudi Jjingo, David Kateete, Oyekanmi Nash, Dorothea Nitsch, Moffat Nyirenda, Dipender Gill, Eleftheria Zeggini, Harald Grallert, Annette Peters, Tinashe Chikowore, Chiara Batini, Opeyemi Soremekun, Segun Fatumo

**Affiliations:** ^1^ The African Computational Genomics (TACG) Research Group, MRC/UVRI and LSHTM Uganda Research Unit, Entebbe, Uganda; ^2^ Department of Immunology and Molecular Biology, School of Biomedical Sciences, Makerere University College of Health Sciences, Kampala, Uganda; ^3^ Centre for Genomics Research and Innovation, NABDA/FMST, Abuja, Nigeria; ^4^ Helmholtz Zentrum München, German Research Center for Environmental Health (GmbH), Institute of Epidemiology, Neuherberg, Germany; ^5^ African Center of Excellence in Bioinformatics and Data-Intensive Sciences, Kampala, Uganda; ^6^ Department of Computer Science, Makerere University, Kampala, Uganda; ^7^ Infectious Diseases Institute, Kampala, Uganda; ^8^ Department of Non-Communicable Disease Epidemiology, London School of Hygiene and Tropical Medicine, London School of Hygiene and Tropical Medicine, United Kingdom; ^9^ MRC/UVRI and LSHTM Uganda Research Unit, Entebbe, Uganda; ^10^ Department of Epidemiology and Biostatistics, School of Public Health, Imperial College London, London, United Kingdom; ^11^ Institute of Translational Genomics, Helmholtz Zentrum München – German Research Center for Environmental Health, Neuherberg, Germany; ^12^ TUM School of Medicine, Technical University of Munich and Klinikum Rechts der Isar, Munich, Germany; ^13^ German Center for Diabetes Research (DZD), München-Neuherberg, Neuherberg, Germany; ^14^ Chair of Epidemiology, Institute for Medical Information Processing, Biometry and Epidemiology, Medical Faculty, Ludwig-Maximilians-Universität München, Munich, Germany; ^15^ Department of Medicine, Brigham and Women’s Hospital, Harvard Medical School Boston, Boston, MA, United States; ^16^ Wits Donald Gordon Medical Centre, School of Clinical Medicine, Faculty of Health Sciences, University of the Witwatersrand, Johannesburg, South Africa; ^17^ Department of Population Health Sciences, University of Leicester, Leicester, United Kingdom; ^18^ University Hospitals of Leicester NHS Trust, Leicester, United Kingdom; ^19^ Molecular Bio-computation and Drug Design Laboratory, School of Health Sciences, University of KwaZulu-Natal, Durban, South Africa; ^20^ Precision Healthcare University Research Institute, Queen Mary University of London, United Kingdom

**Keywords:** Type-2 diabetes, blood cell traits, mendelian randomization, Africa, Hematological traits

## Abstract

**Introduction:**

Observational studies have identified associations between hematological traits and type-2 diabetes mellitus (T2D). However, it is difficult to infer causal effects due to the potential of confounding. Our study utilizes the Mendelian randomization (MR) approach to address the above limitation and investigate the causal effect of hematological traits such as white blood cell (WBC), platelets (PLT), and red blood cell (RBC) on T2D in individuals of African ancestry.

**Methods:**

The participating cohorts included participants of African ancestry in the Blood Cell consortium and the Million Veteran Program dataset. Using GWAS summary statistics, we applied a univariable and multivariable Two-sample MR to estimate the causal relationship between hematological traits and T2D.

**Results:**

In the main IVW MR estimates, genetically predicted levels of mean corpuscular hemoglobin concentration (MCHC), mean corpuscular hemoglobin (MCH), and mean corpuscular volume (MCV) were associated with decreased risk of T2D. We also observed a decreased risk of T2D with genetically predicted total WBC count and neutrophil count (NEU), for the WBC traits. The multivariable analysis further supported the direct associations of genetically predicted MCH, MCHC, and MCV levels with a decreased risk of T2D. For the European ancestry, a similar pattern of association was observed for MCH and MCV.

**Discussion:**

These findings indicate that hematological traits may differentially play a role in the development of T2D and be affected by T2D. However, further research is needed to validate and explore the biological pathways and mechanisms involved in these associations.

## Introduction

Type 2 diabetes (T2D) is a disease that causes high blood sugar levels. This multifaceted disorder affects over 400 million people worldwide with severe complications such as heart disease, high blood pressure, stroke, liver disease, etc. (Sanches et al., 2023). While T2D is majorly caused by insulin resistance, type-1 diabetes is caused by immune-mediated loss of pancreatic beta cells thereby leading to insulin deficiency and it is the most common form of diabetes in children (Redondo et al., 2018). Most of the T2D burden can be found in individuals of African ancestry (Motala et al., 2022; Sinclair, 2019). Africa also has the highest burden of infectious diseases (Omosigho et al., 2023). The body system reacts to these diseases via inflammatory responses. They either eliminate the pathogens or facilitate the removal of the damaged tissues (Chen et al., 2018). Cytokines, which are inflammatory chemicals, have been reported to play a role in the dysfunction of the pancreatic beta cells, which are responsible for insulin production (Yang et al., 2010). These can be a possible alternative pathway to T2D and, therefore, greatly impact the risk of T2D in Africa. In contrast, studies have reported that T2D weakens the immune system, making patients more vulnerable to serious and protracted infections (Knapp, 2013; Carey et al., 2018). Determining the primary risk factors for T2D is hard due to the intricate interplay between genetic and environmental factors that characterize the disease etiology (Murea et al., 2012; Mambiya et al., 2019).

Hematological traits are vital indicators describing the blood cells. They are also efficient and quick laboratory diagnostic methods for assessing inflammation, which may occur in the context of infectious diseases or other underlying conditions (Han et al., 2020). These hematological traits include the red blood cell (RBC), which are hemoglobin (HGB), hematocrit (HCT), Mean corpuscular volume (MCV), Mean corpuscular hemoglobin (MCH), Mean corpuscular hemoglobin concentration (MCHC), red cell distribution width (RDW) and red blood cell count (RBC). The white blood cells (WBC) are basophils (BAS), neutrophils (NEU), lymphocytes (LYM), monocytes (MON), and eosinophils (EOS). The platelets (PLT) are platelet count (PLT) and the mean platelet volume (MPV). These traits are associated with T2D risk (Arkew et al., 2022; Jabeen et al., 2013; Shehri, 2017; Thomas et al., 2003). For instance, Arkew *et al.* found that T2D patients had higher total WBC counts, NEU, LYM, EOS, BAS, RDW, PLT, and MPV than the control group (Arkew et al., 2022). In contrast, haemoglobin levels were considerably lower in T2D patients than in controls (Arkew et al., 2022).

These data suggest that there may be a causal association. However, reverse causation and confounding cannot be ruled out due to the study’s observational nature and cross-sectional design. Mendelian randomization (MR) is a statistical method that utilizes single nucleotide polymorphisms (SNP) as genetic instruments to investigate potential causal associations between exposure and outcome (Sanderson et al., 2022; Richmond and Davey Smith, 2022; Soremekun et al., 2022; Fatumo et al., 2021). In this study, we aim to delineate the causal relationship between hematological traits and T2D by undertaking an MR analysis in individuals of African ancestry. This study can help clinicians improve T2D risk prediction and treatment by utilizing heamtological traits to predict individuals at risk of developing T2D.

**FIGURE 1:**
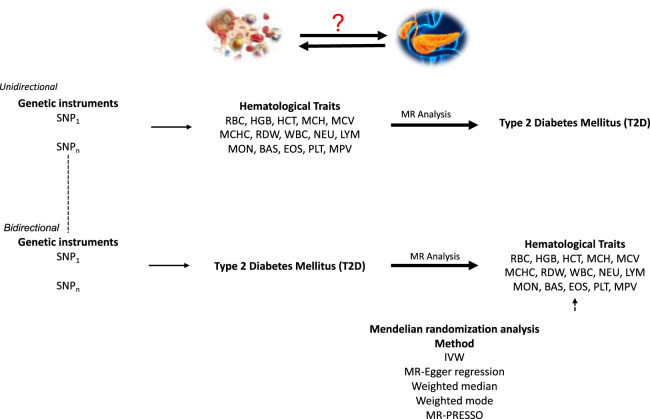
A schematic representation of bi-directional MR analyses highlighting the forward and reverse univariable MR analyses. SNP, single-nucleotide polymorphism; MR, Mendelian Randomization. Assumption 1: Genetic variants used as instruments are associated with the exposures. Assumption 2: Genetic instruments are independent of cofounders that may influence the outcome. Assumption 3: The instruments act on the outcome through their effect on the exposure.

We used publicly available summary statistics from GWAS of hematological traits from the blood cell consortium (Chen et al., 2020) and T2D GWAS summary statistics from the million veteran program (Gaziano et al., 2016). We performed univariable, multivariable, and bidirectional MR to investigate the causal relationship between hematological traits and T2D (Figure 1).

## Materials and methods

### Data source for hematological traits

Hematological traits summary statistics were obtained from the Blood Cell consortium (BCX2). This is a joint effort of various institutions to understand the genetic architecture of hematological traits. Full blood counts were analysed, and subsequent analyses were done using the protocol developed by the consortium. As reported by Chen *et al.*, participants with potential confounding effects to all the hematological traits and extreme blood measures were not included in the GWAS of hematological traits (Chen et al., 2020). Individual studies obtained ethical approval from their respective ethics committee board (Chen et al., 2020).

According to a research article published in 2020, the consortium undertook a transethnic GWAS of 746,667 people of various ancestries, including African, East Asian, European, South Asian, and Hispanic/Latinx populations (Chen et al., 2020) and the meta-analysed data assessed for heterogeneity across individual studies. Different genetic variants were identified to be driving hematological traits in both multi-ancestry and ancestry-specific analysis. We utilized the African ancestry for our analysis. Validation of findings was conducted using a European-ancestry cohort due to the lack of publicly available datasets with sufficient sample size and phenotypic data from African-ancestry populations. This comprises 15 hematological traits (Supplementary Material ST1) from up to 15,171 African Americans and 562,132 Europeans (Chen et al., 2020).

### Data source for Type-2 diabetes

T2D association summary statistics in individuals of African ancestry were obtained from the Million Veteran Program (MVP). This vast biobank comprises consenting veterans at Department of Veteran Affairs facilities (Gaziano et al., 2016; Vujkovic et al., 2020). It is a research program established in 2011 to examine the impact of genetic variation and lifestyle on health and illness. MVP is one of the largest cohorts in the world, with over 875,000 US military veterans of different ethnic groups. Participants in the study gave their permission to take part and to be recontacted by research workers. They also consented to access their electronic health data, gave blood samples for DNA extraction and genotyping, and answered questionnaires regarding their health, lifestyles, and military service. Veterans Affairs Central Institutional Review Board (cCIRB) approved the MVP’s ethical and study protocol (Gaziano et al., 2016). We used the T2D GWAS summary statistics of the MVP from Vujkovic *et al* including 53,445 participants of African ancestry (Ncases = 23,305; Ncontrol = 30,140) (Vujkovic et al., 2020) (Table 1) and 197,066 participants of European ancestry (Ncases = 69,869; Ncontrol = 127,197) (Supplementary Material ST2).

**TABLE 1:** Demographic attributes of the MVP African dataset.

Characteristic	Africans
MVP cohort size, n	53,445 N_cases_ = 23,305; N_control_ = 30,140)
Age at enrolment, mean ± sd	61.7 ± 12.3
Male gender, n (%)	46,603 (87.2%)
BMI (kg/m2), mean ± sd	30.8 ± 6.5
Type 2 Diabetes, n (%)	23,305 (43.6%)

### Genetic instrument selection

To obtain a list of independent genetic instruments from the exposure data, we undertook the steps below: 1) we selected only variants that reached the genome-wide significance threshold (P ≤ 5 × 10^−8^) for association with the exposure under study; 2) we ensured the variants selected in (1) were also present in the outcome data; 3) we pruned the variants for independent SNPs using a linkage disequilibrium correlation coefficient (*r* ^2^) of 0.01 and a distance clumping of ±500 kb. This was done using the African and European reference panel of the 1,000 genome project respectively. We further calculated the proportion of variance in the exposure traits and the instrument strength using the following formula: 2*EAF*(1-EAF)*(beta^2^/var). This was followed by measuring the strength of the instrumental variables (IVs) by calculating the F statistics with the formular; F = (R2*(n - 2))/(1 - R2).

### Statistical analysis

The TwoSampleMR R package (version 0.5.8) (Hemani et al., 2018) and R (version 4.3.2) were utilised to perform MR analyses. We used the inverse variance weighted (IVW) method as the primary analysis. The effect estimate of each genetic variant was meta-analysed using an IVW meta-analysis to determine a single effect of the exposure on the outcome (Lin et al., 2021). We also conducted a bidirectional MR for T2D liability on the hematological traits to further evaluate the direction of causality. To correct for multiple testing, we used the Bonferroni correction method.

### Multivariable mendelian randomization

It has been shown that hematological traits are highly correlated (Chen et al., 2020). Thus, the potential of pleiotropic variants between these traits and their effect on T2D requires proper evaluation. To this end, our analysis used multivariable MR (MVMR) to factor in this potential pleiotropic effect. We ensured that all instruments used fulfill all the conventional instrumental variable assumptions. Conditional F-statistics was carried out to evaluate the strength of the instruments. Modified Cochran’s Q statistic was used to test for horizontal pleiotropy by measuring heterogeneity in causal effect estimates obtained using the IVs (Sanderson et al., 2019). The covariance between the effect of the IVs and exposure was used. The regression-based method implemented in the MVMR function in the TwoSampleMR package was used for this analysis (Burgess and Thompson, 2015).

### Genetic correlation analysis

Genetic correlations are very important as they help to establish the genetic link between traits (Kraft et al., 2020). If genetic factors influencing blood cell traits are genetically linked to T2D, it strengthens the argument for causality. To calculate the SNP heritability (h2) of T2D and hematological traits, as well as the overall genetic correlation (rg) between them, we used cross-trait linkage disequilibrium score regression (LDSC) (Cross-Disorder Group of the Psychiatric Genomics Consortium, 2013).

### Sensitivity analysis

In MR analysis, it is assumed that the genetic instruments are i) associated with the exposure, ii) independent of cofounders, iii) affect the outcome solely through the exposure. Different MR and sensitivity analysis methods were deployed to assess the robustness of our MR inferences and potential violation of the above assumptions. They include MR-Egger (Bowden et al., 2015), weighted median estimator (Bowden et al., 2016), weighted mode estimator (Hartwig et al., 2017), and MR-PRESSO (Mendelian Randomisation Pleiotropy RESidual Sum and Outlier) (Verbanck et al., 2018), SNP scatter plots, single SNP analyses, and leave-one-out analyses. MR-Egger and MR-PRESSO detect and correct for horizontal pleiotropy. This is done by incorporating an intercept term in the regression analysis and identifying and adjusting for outliers among the genetic variants respectively. Single SNP and leave-one-out (LOO) analysis was used to assess the impact of individual genetic variants on the estimated causal effect. LOO works by systematically removing one variant at a time. These help to validate the MR methods. All the genetic instruments were scanned using Phenoscanner (Kamat et al., 2019) to investigate any association between the instrumental variables and any confounding phenotypes. We excluded the instrumental variables associated with confounding phenotypes such as, weight, height (sitting and standing), BMI, etc.

## Results

### Selection of genetic instruments

We selected a total of 428 SNPs as final instruments for MR analysis (Table 2). These SNPs were strongly associated with the hematological traits and are in low linkage disequilibrium with each other. Details of the selected instruments are presented in Table 2 and Supplementary Material ST3–ST5. Traits such as lymphocytes, basophil, and eosinophil did not satisfy these conditions and were not included in the univariate and multivariate analyses. In the bidirectional analysis (T2D as exposure), 16 independent SNPs were used as genetic instruments, to proxy the effect of T2D on hematological traits.

**TABLE 2:** Details of IVs for bidirectional MR analyses of hematological traits and T2D.

Trait	Number of instruments	F-statistics	PVE
RBC	17	57.398	0.116
HGB	5	34.261	0.021
HCT	2	36.014	0.004
MCH	26	97.129	0.293
MCV	29	76.994	0.286
MCHC	16	73.696	0.138
RDW	9	48.708	0.039
WBC	134	56.479	0.787
NEU	152	69.938	1.018
MON	14	47.878	0.088
PLT	11	249.848	0.059
MPV	10	468.832	0.107
T2D	16	72.345	0.171

PVE, percentage variance explained.

### Genetic correlation analysis

Using LDSC, the SNP heritability was estimated to be 0.1414 (se = 0.0276) for T2D. We found that there existed a significant genetic correlation between T2D and HCT (rg = −0.26 p = 0.03), HGB (rg = −0.24 p = 0.03), EOS (rg = 0.44 p = 0.05), PLT (rg = 0.35 p = 0.002) in Africans. We also observed a significant genetic correlation between T2D and HGB (rg = 0.07 p = 0.0008), HCT (rg = 0.06 p = 0.005), WBC (rg = 0.09 p = 2.3E-6), NEU (rg = 0.06 p = 0.003), LYM (rg = 0.09 p = 4.19E-6), MCH (rg = −0.04 p = 0.01), MCV (rg = −0.06 p = 0.0006) and RBC (rg = 0.07 p = 6.19E-5) (Supplementary Material ST19).

### MR results of genetically proxied hematological trait levels on T2D

**FIGURE 2:**
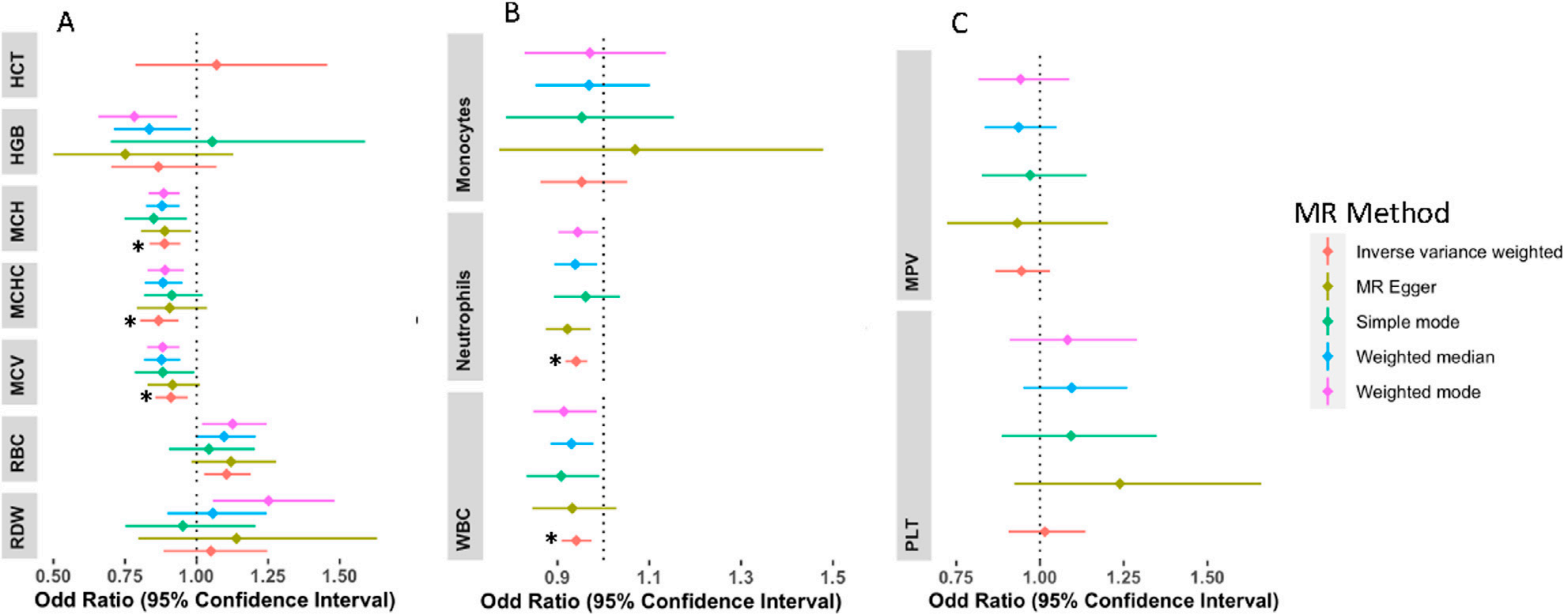
Forest plots of the odd ratios (OR) and 95% confidence interval between red blood cell traits and T2D **(A)**, white blood cell traits and T2D **(B)**, and platelets and T2D **(C)**. NB: Other sensitivity analyses were not included for HCT because this sensitivity analysis had a large confidence interval; however, it was in the same direction as the IVW analysis. *Associations that remained significant after multiple testing correction.

Concerning the association of hematological traits with T2D, odds ratios (ORs) and 95% confidence intervals (CIs) from IVW MR are presented in Figure 2. Genetically predicted MCHC, MCH, and MCV levels were associated with decreased risk of T2D with an OR_IVW_ per SD change of 0.870 (95% CI: 0.806–0.940; P_IVW_ = 0.0004), 0.895 (95% CI: 0.846–0.947; P_IVW_ = 0.0001) and 0.905 (95% CI: 0.851–0.963; P_IVW_ = 0.0015) respectively. Across the sensitivity analyses, the associations exhibited similar effect sizes and directions (Supplementary Material ST4). Genetically high levels of RBC were associated with an increased risk of T2D with OR_IVW_ of 1.106 per SD change in RBC levels (95% CI: 1.017–1.203; P_IVW_ = 0.0180), however, this was not significant after adjusting for multiple testing. There was no evidence of a causal relationship between genetically predicted HCT [OR = 1.069(CI: 0.786–1.456, P = 0.667)], HGB [OR = 0.866(CI: 0.701–1.070, P = 0.183)], and RDW [OR = 1.050(CI: 0.884–1.247, P = 0.576)] with risk of T2D. These associations remained the same in all other sensitivity analyses (Supplementary Material ST6).

For white blood cell traits, genetically predicted WBC and neutrophil levels were associated with decreased risk of T2D, WBC [OR = 0.941 (CI: 0.908–0.975, P = 0.0007] and neutrophils [OR = 0.941 (CI: 0.917–0.965, P = 2.88E-06] on T2D. However, no association was detected in monocytes (Supplementary Material ST6). Similarly, no evidence of association detected for platelets and T2D risk; PLT [OR = 1.014(CI: 0.906–1.134, P = 0.800)] and MPV [OR = 0.9433(CI: 0.860–1.012, P = 0.097)]. All sensitivity analyses were in the same direction as the main (IVW) analysis except in MPV which had MR-Egger in the opposite direction (Supplementary Material ST6). The MR-PRESSO and other sensitivity analyses are detailed in supplementary (Supplementary Material ST7–ST9 and Supplementary Figures 1, 2).

### MR results of genetically proxied T2D on hematological traits

**FIGURE 3:**
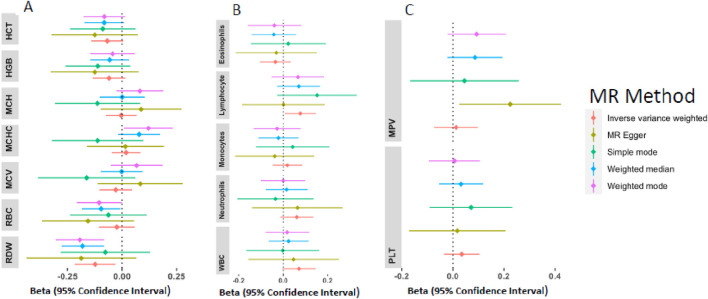
Forest plots of the beta estimates and 95% confidence interval between T2D and red blood cell traits **(A)**, T2D and white blood cell traits **(B)**, and T2D and platelets **(C)**.

Estimates of the genetically predicted T2D liability on hematological traits were calculated by the IVW method (Figure 3). Genetically predicted T2D risk was associated with a lower level of RDW [B = −0.123(CI: 0.217–0.030, P = 0.009)]; however, after correcting for multiple testing, the association was not significant. There were no evidence of MR association between T2D liability and HCT [B = −0.068(CI: 0.141-0.005, P = 0.067)], HGB [B = −0.059(CI: 0.135-0.0150, P = 0.117], MCH [B = −0.003(CI: 0.075-0.067, P = 0.917)], MCHC [B = 0.018(CI: 0.047-0.084, P = 0.587)], MCV [B = −0.028(CI: 0.1030-0.046, P = 0.463)], and RBC [B = −0.023(CI: 0.059–0.977, P = 0.579)] (Supplementary Material ST11).

For the WBC, genetically predicted T2D risk was associated with elevated levels of lymphocytes [B = 0.076(CI: 0.006–0.146, P = 0.032)]; however, this was not significant after adjusting for multiple testing. No significant association was detected between T2D and NEU [B = 0.061(CI: 0.014-0.135, P = 0.109)], WBC [B = 0.070(CI:-0.0049-0.145, P = 0.067)], MON [B = 0.018(CI:-0.047-0.085, P = 0.576)], EOS [B = −0.035(CI:-0.104-0.034, P = 0.322)], and BAS [B = 0.002(CI:-0.075-0.081, P = 0.946)] (Supplementary Material ST11). T2D showed no significant effect on platelet traits (Supplementary Material ST11). Genetic instrument information for T2D liability are shown in Supplementary Material ST10.

### Multivariable mendelian randomization

**FIGURE 4:**
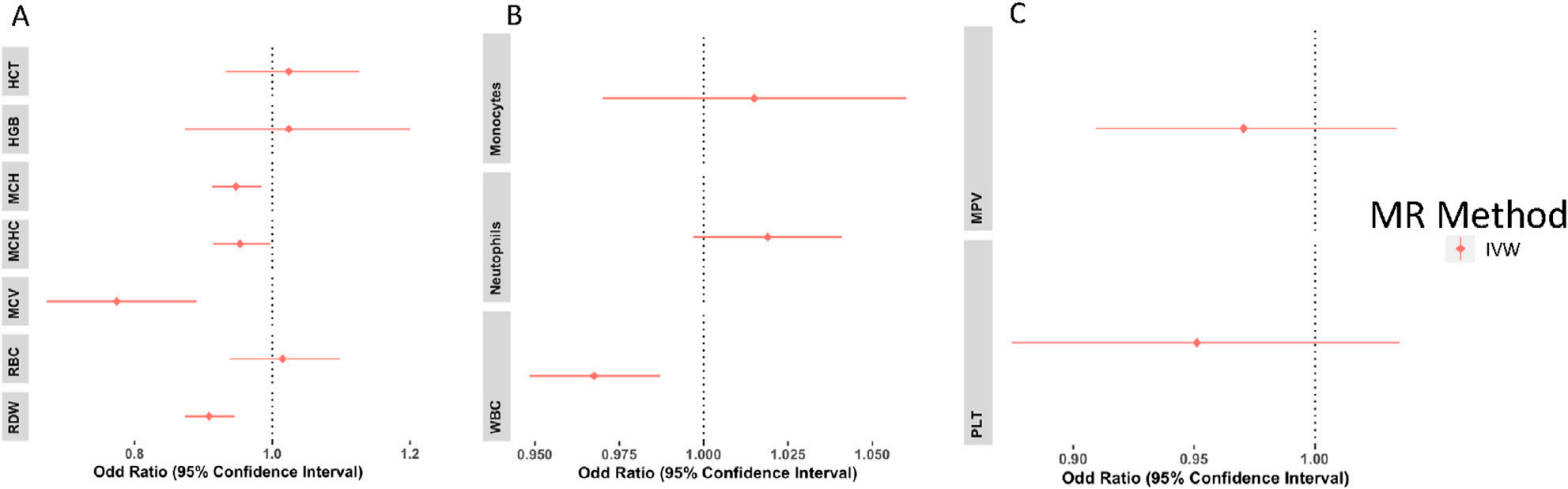
Multivariable analysis forest plots of the odd ratios (OR) and 95% confidence interval between red blood cell traits and T2D **(A)**, white blood cell traits and T2D **(B)**, and platelets and T2D **(C)**.

The results of the multivariable analyses generally mirrored those of the univariable analyses. As shown in Figure 4, genetically predicted MCH, MCHC, RDW, and MCV were significantly associated with decreased risk of T2D using the IVW estimator with an OR of 0.947(CI: 0.913–0.984, P = 0.005), 0.953 (CI: 0.914–0.997, P = 0.035), 0.908(CI: 0.873–0.946, P= <0.001) and 0.774(CI: 0.672–0.890, P= <0.001), respectively. Although the causal association detected between genetically predicted RBC and T2D risk in the univariable analysis was not significant in the multivariable analysis [OR = 1.015 (CI: 00.938–01.098, P= <0.71)], it showed the same direction of effect on T2D. MCH, MCV, and MCHC have conditional F-statistics (Supplementary Material ST13) above the conventional threshold of 10. This signifies that they are strong enough to be used as an instrument for MVMR. RBC, HGB, HCT, and RDW have conditional F-statistics below 10, signifying that the instruments are weak. No association was observed between genetically predicted HGB and HCT, and risk of T2D (Supplementary Material ST12).

Genetically predicted lower levels of WBC were associated with decreased risk of T2D [OR = 0.967(CI: 0.948–0.987, P = 0.001)]. Neutrophils, monocytes, PLT, and MPV showed no significant association with T2D (Figure 4, Supplementary Material ST12). WBC, NEU, MON, PLT, and MPV all have conditional F-statistics above 10, thereby validating their strength as an instrument. The Cochran’s *Q* test suggests that there was no statistically significant evidence of pleiotropy in the estimated causal effects across the MVMR IVs for the WBC and PLT, however, a significant pleiotropy was observed for the RBC (Supplementary Material ST13).

### MR results of genetically proxied hematological trait levels on T2D in Europeans

**FIGURE 5:**
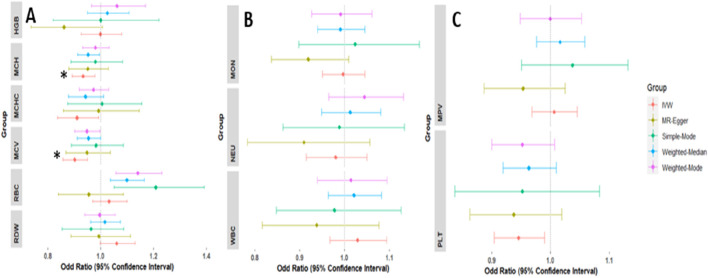
Forest plots of the odd ratios (OR) and 95% confidence interval between red blood cell traits and T2D **(A)**, white blood cell traits and T2D **(B)**, and platelets and T2D **(C)** in the European population. *Associations that remained significant after multiple testing corrections.

We validated our findings in the African ancestry population using European ancestry individuals. The association of hematological traits with T2D, odds ratios (ORs), and 95% confidence intervals (CIs) from IVW MR are presented in Supplementary Material S17 and Figure 5.

Genetically predicted MCH and MCV levels were significantly associated with decreased risk of T2D with an OR_IVW_ per SD change of 0.934 (95% CI: 0.891–0.98; P_IVW_ = 0.003) and 0.902 (95% CI: 0.857–0.949; P_IVW_ = 0.00008) respectively. Genetically high levels of MCHC were associated with an increased risk of T2D with OR_IVW_ of 0.911 per SD change in MCHC levels (95% CI: 0.836–0.992; P_IVW_ = 0.0032), however, this was not significant after adjusting for multiple testing. There was no evidence of a causal relationship between genetically predicted HGB [OR = 0.999(CI: 0.926–1.078, P = 0.990)], HCT [OR = 1.044(CI: 0.962–1.125, P = 0.254)] and RBC [OR = 1.032(CI: 0.970–1.247, P = 1.099)] with risk of T2D. Across all sensitivity analyses, the associations exhibited similar effect sizes and directions (Supplementary Material ST17).

For white blood cell traits, contrary to our observation in the African ancestry, there was no association of MON [OR = 0.996 (CI: 0.950–1.045, P = 0.894], NEU [OR = 0.980 (CI: 0.914–1.050, P = 0.572] and WBC [OR = 1.029(CI: 0.967–1.094, P = 0.360)] on T2D (Supplementary Material ST17). In addition, there was decreased risk association detected for platelets, PLT [OR = 0.946(CI: 0.904–0.989, P = 0.015)] and but no evidence of association for MPV [OR = 1.006(CI: 0.968–1.045, P = 0.746)] with risk of T2D. All sensitivity analyses were in the same direction as the main (IVW) analysis except in MPV, which had MR-Egger in the opposite direction (Supplementary Material ST18). The MR-PRESSO and other sensitivity analyses, including Single SNP and leave-one-out analysis, are detailed in Supplementary Material ST18. Genetic instruments are shown in Supplementary Material ST14–ST16.

## Discussion

In this MR study, we examined the relationship between hematological traits and T2D risk in African ancestry individuals. Our results showed that genetically higher levels of MCHC, MCH, and MCV were associated with decreased risk of T2D. MCH and MCV levels play major roles in erythropoiesis and hematopoiesis (Vega-Sánchez et al., 2020). Optimal production of the RBC could indirectly impact metabolic pathways and potentially lead to a lower risk of T2D. MCHC level which reflects the concentration of hemoglobin in red blood cells is directly influenced by iron levels (Choy et al., 2023). Insulin sensitivity and the metabolism of iron are closely linked (Vaquero et al., 2020), therefore higher MCHC levels might suggest more efficient iron metabolism, potentially contributing to improved insulin sensitivity and reduced T2D risk.

This indicates that individuals who are genetically predisposed with elevated MCHC, MCH, and MCV may exhibit a decreased risk of T2D. The effect size shows approximately 4.7%, 5%, and 23% reduction in the risk of developing T2D per SD increase respectively. The consistency across multiple sensitivity analyses increases our confidence in the observed causal relationship. This suggests a potential role of these RBC traits in glucose homeostasis, and it also provides insights into novel markers that may be utilized for early detection of T2D. There was no evidence of associations between genetically determined HCT, HGB, and RDW with T2D. This trend can also be seen in the multiple sensitivity analysis carried out.

There was a decreased between WBC and neutrophil on T2D risk for white blood cell traits, while no evidence of associations was detected for monocytes. These WBCs play a great role in the immune response. A well-regulated and balanced immune system helps modulate low-grade inflammation associated with insulin resistance and T2D (Tsalamandris et al., 2019; Galicia-Garcia et al., 2020). This indicates that individuals genetically predisposed to elevated WBC and neutrophils may exhibit a decreased risk of T2D. The effect sizes for both show approximately a 5.9% reduction in the risk of developing T2D per SD increase. This explains the importance of immune system modulation in mitigating T2D susceptibility. There was no MR evidence of association detected for PLT with T2D risk. Additionally, the study showed that genetically predicted T2D was not associated with any of the hematological traits. This could be because T2D is a complex disorder or because the sample size does not allow for the detection of any statistical association.

Comparing our findings in the European population, we observed that genetically predicted MCH and MCV levels were significantly associated with decreased risk of T2D. Genetically high levels of MCHC were also associated with a decreased risk of T2D, however, this was not significant after adjusting for multiple tests. No evidence of a causal relationship was seen in other RBC traits, and this validates our discovery in the African population. However, for the white blood cell traits, we observed an opposite effect in the Europeans. There was no association of MON, NEU, and WBC on T2D, unlike the African population. This can be explained by genetic diversity observed among different populations, considering that immune responses are shaped by exposure to infections (Cheung et al., 2023; Serre and Pääbo, 2004). The prevalence of different infections in different populations can also influence the observed association.

Observation and experimental studies have demonstrated that blood cell trait and T2D are associated (Arkew et al., 2022; Wang et al., 2021; Nwagu, 2015). Studies have further confirmed that morphological and concentration level changes in blood cell traits are common in people with diabetics (Wautier and Wautier, 2013; Biadgo et al., 2016; Nada, 2015; Malandrino et al., 2012). The findings from this Mendelian randomization study on the association between blood cell traits and T2D are consistent with previous observation studies on this topic. A study by Waggiallah *et al* reported that people with diabetes have lower red cell indices, such as MCV, MCH, and MCHC (Waggiallah and Alzohairy, 2011). Our forward MR analysis results are consistent with other studies that have found an association between WBC count and T2D (Kheradmand et al., 2021; Kashima et al., 2019; Vozarova et al., 2002; Twig et al., 2013; Schmidt et al., 1999; Gkrania-Klotsas et al., 2010; Freeman et al., 2001; Duncan et al., 2003). For instance, neutrophil and lymphocyte count in participants of the atherosclerosis risk in community research were associated with T2D (Schmidt et al., 1999) and the same with the EPIC-Norfolk study (Gkrania-Klotsas et al., 2010).

However, our study has some limitations. One limitation of our study is using a European-ancestry cohort for validation instead of an ancestrally similar African-ancestry cohort. This choice was necessitated by the unavailability of African-ancestry datasets with adequate sample sizes and various hematological phenotypes. However, this approach provides valuable insights into shared genetic signals across populations. Also, RBC lifespan has been noted to be affected by HbA1c, and T2D has also been diagnosed using HbA1c level (Abass et al., 2017). Hence, there might be an intrinsic association between RBC and T2D. We performed an MR analysis between RBC and another glucose intolerance-associated trait (2-h glucose level) to validate our findings. HCT, MCH, MCHC, MCV, and RBC have the same direction of effect when compared to T2D, even though, none was significantly associated. This might indicate a potential trend or a suggestive association and, therefore, requires further validation.

In addition, the individuals included in the blood cell consortium and the GWAS of T2D may have some participants in common. This is because the blood cell consortium is a meta-analysis of different cohorts, including individuals from the MVP. We acknowledge this might introduce some bias; however, the sample overlap is not large enough to alter the MR estimates. We also selected strong instruments in the main univariable analysis, as indicated by the F statistics. In the multivariable analysis, we observed low-conditioned F-statistics for red blood cell traits such as RBC, HCT, HGB, and RDW. This is probably due to the high correlation that exists between these traits; hence, testing the independent effect is difficult. To this end, the result of the multivariable should be interpreted with caution.

Pleiotropy is an important issue worth considering in MR analysis, as effects of genetic instruments that are pleiotropic but are not mediated by exposure on the outcome could lead to biased causal estimates (Davey Smith and Hemani, 2014). We, therefore, conducted a wide range of sensitivity analyses, each with its assumptions regarding the pleiotropic effects of the instruments, to reduce the risk of bias (Bowden et al., 2015; Bowden et al., 2016; Hartwig et al., 2017). In addition, this study may have been limited by the relatively small sample sizes of the blood cell traits and T2D. These samples are, however, the largest samples available in individuals of African ancestry, which the authors have access to at the time of the analysis.

Of note is the categorization of this data in terms of ancestry. Most of the data are from African Americans, most especially the MVP cohort; this may not be a true representation of Africans as African-American’s genomes do not capture Africa’s genetic diversity. Variations in blood cell trait measurement methods, performance assays across contributing cohorts in the BCX consortium, and changes over time could impact the GWAS used to estimate causal effects in our MR analysis.

Our MR study adds to the body of evidence supporting the link between hematological traits and T2D risk in people of African ancestry. Despite the mentioned limitations, our results highlight the potential contributions of blood cell traits such as white blood cell count and red blood cell count, to the emergence of T2D. For future studies, we aim to integrate our MR findings with omics data (transcriptomics and proteomics) to elucidate the biological pathway connecting these hematological traits and T2D.

## Data Availability

Publicly available datasets were analyzed in this study. This data can be found here: http://www.mhi-humangenetics.org/en/resources/.

## Supplementary material

Supplementary figure 1 Scatter plots illustrating the Mendelian Randomization (MR) analysis for six red blood cell (RBC) related traits. Each plot shows the SNP effect on exposure (X-axis) vs. SNP effect on outcome (Y-axis) with different MR methods, including Inverse Variance Weighted (IVW), MR Egger, Weighted Median, Weighted Mode, and Simple Mode.

Supplementary figure 2 Scatter plots illustrating the Mendelian Randomization (MR) analysis for five white blood cell (WBC) and platelets (PLT) related traits. Each plot shows the SNP effect on exposure (X-axis) vs. SNP effect on outcome (Y-axis) with different MR methods, including Inverse Variance Weighted (IVW), MR Egger, Weighted Median, Weighted Mode, and Simple Mode.

## Abbreviations

T2D, Type 2 Diabetes Mellitus; RBC count, red blood cell count; HGB, haemoglobin concentration; HCT, hematocrit; MCH, mean corpuscular haemoglobin; MCV, mean corpuscular volume; MCHC, mean corpuscular haemoglobin concentration; RDW, RBC distribution width; MR, Mendelian Randomization; IVW, Inverse-variance weight; IV, Instrumental Variable.
