# Supplementary material for: Mendelian randomization study highlights the role of hematological traits on Type-2 diabetes mellitus in African ancestry individuals
Source: Front Pharmacol. 2025 Mar 31;16:1436972. doi: 10.3389/fphar.2025.1436972 (PMC11994964; doi:10.3389/fphar.2025.1436972)
Supplement: Supplementary file 1 [file DataSheet1.pdf]

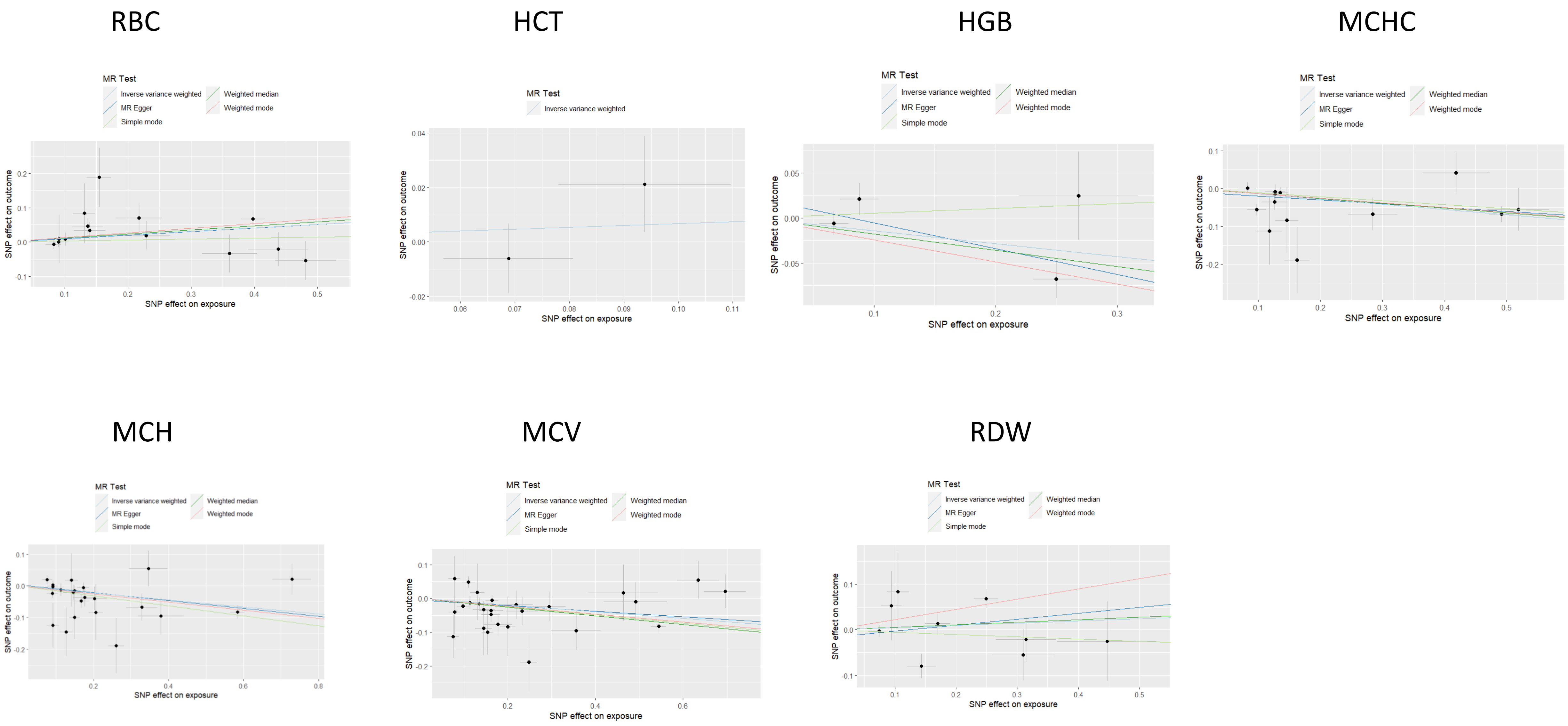

Figure 1: SNP Scatter Plot for the RBC

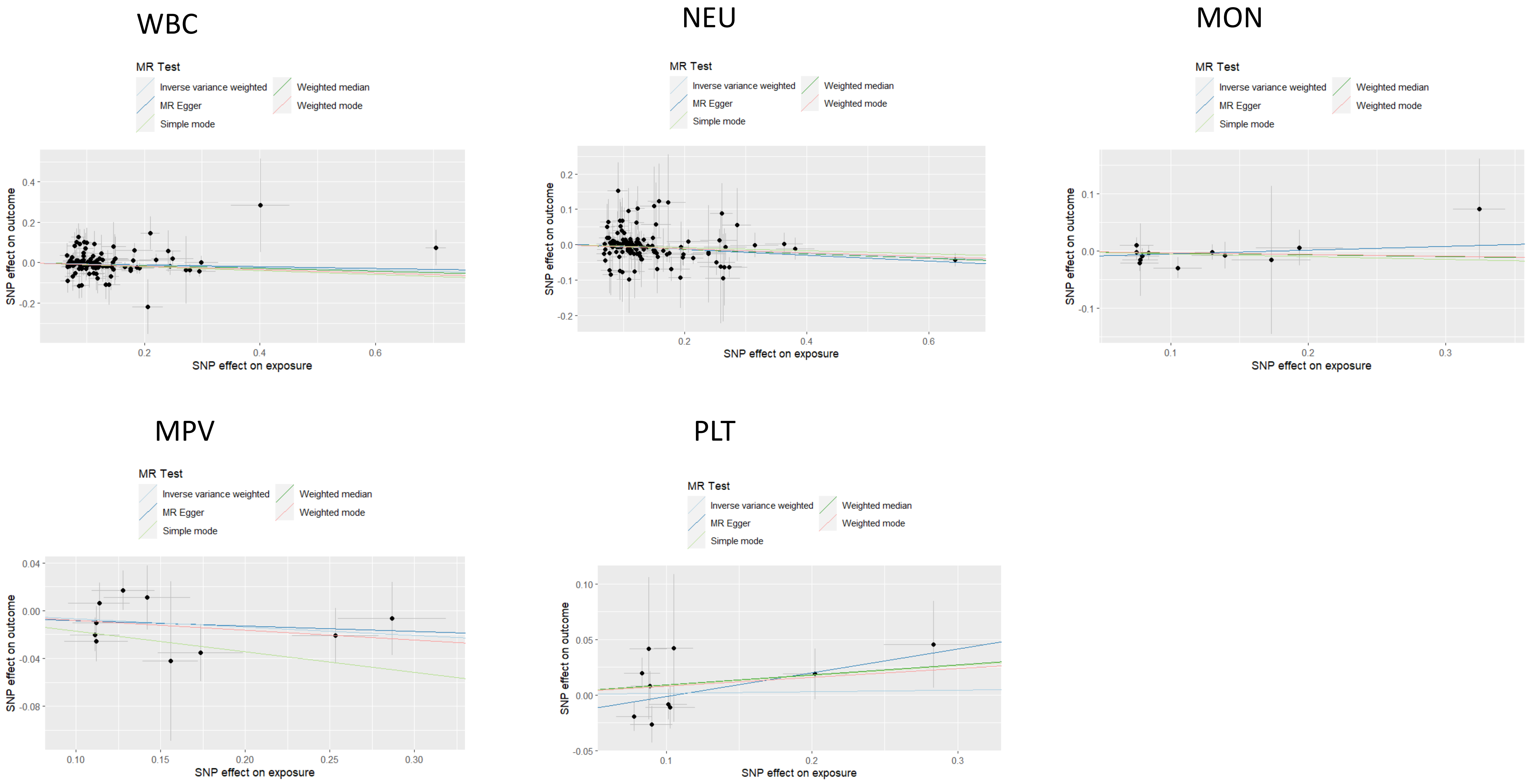

Figure 2: SNP Scatter Plot for the WBC and PLT
